# Proteostasis in Cerebral Small Vessel Disease

**DOI:** 10.3389/fnins.2019.01142

**Published:** 2019-11-15

**Authors:** Christof Haffner

**Affiliations:** Institute for Stroke and Dementia Research, Klinikum der Universität München, Ludwig-Maximilians-Universität München, Munich, Germany

**Keywords:** CADASIL, CARASIL, cerebral amyloid angiopathy, HTRA1, Notch3, proteomics, chaperone

## Abstract

Maintaining the homeostasis of proteins (proteostasis) by controlling their synthesis, folding and degradation is a central task of cells and tissues. The gradual decline of the capacity of the various proteostasis machineries, frequently in combination with their overload through mutated, aggregation-prone proteins, is increasingly recognized as an important catalyst of age-dependent pathologies in the brain, most prominently neurodegenerative disorders. A dysfunctional proteostasis might also contribute to neurovascular disease as indicated by the occurrence of excessive protein accumulation or massive extracellular matrix expansion within vessel walls in conditions such as cerebral small vessel disease (SVD), a major cause of ischemic stroke, and cerebral amyloid angiopathy. Recent advances in brain vessel isolation techniques and mass spectrometry methodology have facilitated the analysis of cerebrovascular proteomes and fueled efforts to determine the proteomic signatures associated with neurovascular disease. In several studies in humans and mice considerable differences between healthy and diseased vessel proteomes were observed, emphasizing the critical contribution of an impaired proteostasis to disease pathogenesis. These findings highlight the important role of a balanced proteostasis for cerebrovascular health.

## Introduction

Proteome homeostasis or proteostasis refers to the maintenance of all proteins of an organism in a conformation, concentration, and location that is required for their correct function (Balch et al., 2008). Safeguarding proteostasis under a wide range of environmental and metabolic conditions is a fundamental task of cells and tissues and ensured by a proteostasis network, which controls protein synthesis, folding, trafficking, and degradation/clearance (Hipp et al., 2019). The surveillance of protein folding is a particularly challenging problem, since incompletely folded and misfolded proteins are common byproducts of the cell metabolism and represent a considerable risk due to their high aggregation potential. Molecular chaperones counteract aggregation by facilitating the folding/refolding of proteins on a physiological timescale or by initiating the degradation of terminally misfolded proteins through the ubiquitin–proteasome or the autophagosome–lysosome system (Hartl et al., 2011). Chaperones shield hydrophobic amino acid residues and unpaired β-strands of incorrectly folded proteins from the aqueous cellular environment and abolish the formation of intermolecular contacts between non-native protein states. Yet, excessive protein misfolding due to increased environmental stress or genetic mutations is a frequent cause of pathological conditions (Wyatt et al., 2013; Chiti and Dobson, 2017). These proteinopathies are characterized by an overload and failure of the proteostasis network and a subsequent predominance of aggregation-mediated proteotoxicity over aggregate clearance inside and/or outside the cell. Prominent examples are degenerative disorders of the brain associated with protein aggregates adopting well-ordered structures referred to as amyloid. The progressivity of these conditions with age is likely linked to the age-dependent decline of the proteostasis network capacity (Labbadia and Morimoto, 2015; Hipp et al., 2019). How protein aggregates cause toxicity and cellular dysfunction is only partially understood, but once aggregation has been initiated a further collapse in proteostasis is likely to occur due to the sequestration of chaperones and other proteostasis components into pathologic inclusions (Balchin et al., 2016).

While the molecular details of intracellular proteostasis have been investigated extensively, our understanding of the mechanisms maintaining proteostasis in the extracellular space is limited. The discovery of a small but growing number of constitutively secreted chaperones has advanced our knowledge about processes counteracting extracellular proteotoxicity (Wyatt et al., 2013). These chaperones share functional similarities with the family of intracellular small heat shock proteins in that they recognize targets via exposed hydrophobic regions and serve as sensors as well as disposal mediators of misfolded proteins. However, they lack ATPase activity and cannot refold proteins, limiting their role in safeguarding against proteotoxicity to protein degradation and aggregate removal. The clearance of misfolded proteins from the brain parenchyma is a particular challenging task and believed to occur to a substantial extent via transport along glymphatic/perivascular routes (Morris et al., 2016; Rasmussen et al., 2018). The impairment of this process is likely to facilitate protein aggregation and deposition within the brain vasculature and to promote the development of cerebrovascular pathologies (Carare et al., 2013; Weller et al., 2015). Two important examples are cerebral amyloid angiopathy (CAA), an important cause of intracerebral hemorrhage, and cerebral autosomal-dominant arteriopathy with subcortical infarcts and leukoencephalopathy (CADASIL), the most prevalent monogenic form of lacunar (ischemic) stroke in adults. Both are characterized by excessive protein accumulation in brain vessel walls highlighting the importance of a balanced extracellular proteome for brain health.

## Cerebral Small Vessel Disease

Cerebrovascular disease resulting in vascular cognitive impairment (VCI)/vascular dementia (VD) is increasingly recognized as important cause of dementia in the elderly and has become a major challenge for aging societies (Iadecola, 2013). It can occur in “pure” form without concomitant cerebral pathologies or, much more frequently, in “mixed” form together with pathologies of neurodegenerative disorders, most prominently Alzheimer’s disease (AD) or Parkinson’s disease (Jellinger, 2013). Cerebral small vessel disease (SVD), a major cause of VCI, describes a heterogeneous group of conditions affecting small arteries, arterioles, capillaries and sometimes venules of the brain and comprises CAA as well as non-amyloid arteriopathies (Pantoni, 2010; Kalaria, 2018; Vinters et al., 2018). CAA is clinically characterized by microbleeds, microinfarcts and hemorrhagic stroke and histologically clearly defined by the accumulation of insoluble amyloid-β (Aβ) peptides in the walls of small, mainly cortical and leptomeningeal vessels (Charidimou et al., 2017). In contrast, non-amyloid SVD results in both lacunar stroke as well as intracerebral hemorrhage and encompasses common sporadic pathologies such as small vessel arteriosclerosis, lipohyalinosis and arteriolosclerosis as well as rare hereditary syndromes (Pantoni, 2010; Wardlaw et al., 2019). Histopathological features include concentric hyaline vessel wall thickening, luminal stenosis, accumulation of amorphous proteinaceous material and plasma proteins in the vessel matrix as well as loss of mural cells [vascular smooth muscle cells (VSMC), pericytes] from the tunica media (Grinberg and Thal, 2010; Pantoni, 2010). Some insight into the underlying molecular mechanisms has been gained from hereditary SVD forms with defined genetic etiology and the analysis of the biology and physiology of the underlying genes (Haffner et al., 2016; Tan et al., 2017; Yamamoto and Ihara, 2017). The following sections provide an overview of the molecular etiologies and pathological features of the SVD forms relevant for the topic of this review.

## Cadasil and Carasil

Cerebral autosomal-dominant arteriopathy with subcortical infarcts and leukoencephalopathy is the most common heritable cause of stroke and VCI in adults (Chabriat et al., 2009; Joutel, 2011). It is a progressive neurological syndrome associated with ischemic events and cognitive decline affecting young or middle-aged individuals and leading to a terminal stage of being bedridden and demented within a mean of 25 years. In magnetic resonance imaging (MRI) patients display the typical signs of chronic small artery disease of the brain such as white matter damage, lacunar infarcts and dilated perivascular spaces (Chabriat et al., 2009). Histological analysis of vessels shows a marked thickening of the walls of small and medium-sized, leptomeningeal and penetrating arteries, luminal stenosis and the presence of ultrastructural, non-amyloid protein deposits termed granular osmiophilic material (GOM) in the extracellular space of the tunica media (Kalimo et al., 2002; Tikka et al., 2014). Although a structural and functional impairment of mural cells of the vessel wall has been reported (Ghosh et al., 2015; Capone et al., 2016b), many aspects of the pathophysiological processes leading to vessel degeneration and dysfunction are still unknown. CADASIL is caused by mutations in the *NOTCH3* gene (Chabriat et al., 2009; Rutten et al., 2014), which encodes a signaling receptor essential for VSMC survival, blood vessel integrity, blood-brain barrier function and regulation of vascular tone (Henshall et al., 2015; Kofler et al., 2015). Notch3 is a large, single-pass transmembrane protein with an extracellular domain (Notch3^ECD^) mainly consisting of 34 tandem epidermal growth factor (EGF)-like repeats and an intracellular domain acting as a transcriptional coactivator when proteolytically released from the membrane-bound receptor upon ligand-mediated activation (Siebel and Lendahl, 2017). Although a loss of Notch3 function cannot be completely ruled out as driving force for CADASIL pathogenesis (Machuca-Parra et al., 2017; Coupland et al., 2018), a gain of toxic function is currently widely accepted as disease mechanism (Joutel, 2011; Haffner et al., 2016). Typical CADASIL mutations are missense variants in the Notch3^ECD^ causing a gain or loss of cysteine residues and resulting in a disruption of the highly conserved disulfide bond pattern characteristic for EGF repeats (Chabriat et al., 2009). As a consequence, mutant receptors are likely to engage via unpaired sulfhydryl groups in abnormal intermolecular interactions leading to Notch3^ECD^ aggregation (Duering et al., 2011) and the formation of focal protein deposits corresponding to the ultrastructural GOM (Joutel, 2011). Notch3^ECD^ aggregates are believed to confer toxicity by dysregulating ECM homeostasis, but the underlying molecular processes are largely unknown (Joutel et al., 2016).

Another monogenic SVD related to CADASIL is CARASIL (cerebral autosomal-recessive arteriopathy with subcortical infarcts and leukoencephalopathy), a rare familial form of non-hypertensive SVD with an age of onset in the second or third decade of life (Fukutake, 2011; Nozaki et al., 2014). As the acronym implies, it shows an overlap in clinical and histopathological features with CADASIL, with cardinal neurological features being early onset lacunar stroke primarily in the basal ganglia or brainstem, extensive white matter abnormalities and premature cognitive decline. Clinically, it can be differentiated from CADASIL mainly due to the recessive inheritance pattern and the extraneurological symptoms such as premature baldness and spondylosis. Histological analysis typically reveals extensive pathological alterations of the cerebral vasculature including vessel wall thickening, vessel lumen narrowing, elastic lamina splitting and VSMC loss (Oide et al., 2008; Tikka et al., 2014). CARASIL is caused by mutations in the conserved serine protease HTRA1 (high temperature requirement A1) through a loss-of-function mechanism attributed to a reduction of its proteolytic capacity or to mRNA instability (Hara et al., 2009; Shiga et al., 2011; Beaufort et al., 2014; Nozaki et al., 2014). Heterozygous HTRA1 mutations were further found to cause a dominant, late-onset form of SVD with a milder phenotype (Verdura et al., 2015), but whether this condition results from haploinsufficiency or a dominant-negative effect is an unresolved issue (Nozaki et al., 2016). HTRA1 is a primarily secreted protease and a member of a conserved protein family, which has well documented roles in cellular quality control processes in bacteria and plants (Clausen et al., 2011). The function of human HTRA1 is less clear, but findings over the last decade including the proteomic studies described below indicate a crucial role in balancing the extracellular proteome.

## Cerebral Amyloid Angiopathy

Cerebral amyloid angiopathy is a brain condition frequently associated with Alzheimer’s disease (AD) and characterized by the accumulation of Aβ peptides in the cerebral vasculature (Charidimou et al., 2017). Its major clinical presentations are spontaneous intracerebral hemorrhage, cognitive impairment and dementia, with MRI signatures including multiple, strictly lobar cerebral microbleeds, white matter hyperintensities, cortical microinfarcts and enlarged perivascular spaces. Despite its close molecular and clinical relationship with AD, CAA remains clinically distinct (Viswanathan and Greenberg, 2011). The overall cognitive profile of CAA patients, which primarily comprises executive dysfunction and impaired processing speed with relatively preserved episodic memory, is more similar to that seen in classic VCI (Charidimou et al., 2017). The vasculopathic changes include fibrinoid necrosis, VSMC loss and vessel wall thickening (Attems et al., 2011), degenerative processes also observed in ischemic SVD. Nevertheless, CAA differs from non-amyloid SVD in various aspects: First, it preferentially affects small arterioles and capillaries of the leptomeninges and cerebral cortex, without necessarily involving white matter vessels; second, it lacks association with hypertension, arteriosclerosis and other common vascular risk factors; and third, it is mainly observed in posterior lobar brain regions (especially the occipital lobes), whereas basal ganglia and brainstem are usually spared (Reijmer et al., 2016). Two different types of CAA can be distinguished, with type 1 (capillary CAA) being characterized by Aβ deposition in capillaries (with or without involvement of larger vessels), and type 2 showing Aβ pathology exclusively in leptomeningeal and cortical arteries (Attems et al., 2011). Current evidence suggests that cerebrovascular Aβ accumulation is largely driven by a failure to efficiently clear parenchymal Aβ via perivascular routes (Carare et al., 2013; Weller et al., 2015). While the molecular mechanisms leading to the arteriopathy are largely unknown, alterations in ECM structure and composition, similar to ischemic SVD, likely play a critical role (Morris et al., 2014).

## Proteomic Studies in SVD

Liquid chromatography/tandem mass spectrometry (LC-MS/MS) has become an invaluable technology for global proteomic analyses of tissues under physiological and pathophysiological conditions, allowing the elucidation of disease-relevant molecular processes and pathways (Aebersold and Mann, 2016). In recent years a number of proteomic studies have been conducted on cerebrovascular tissue isolated from CADASIL and CAA post-mortem samples to determine the disease-specific proteome profiles and revealed a distinct set of proteins enriched in both disorders with unexpected consistency (Table 1). In two of the four CADASIL studies, laser-capture microdissection was used to isolate brain artery material, resulting in the identification of a relatively low total number of proteins (Arboleda-Velasquez et al., 2011; Nagatoshi et al., 2017). Arboleda-Velasquez et al. (2011) used subcortical white matter tissue from two patients and two healthy control individuals and semiquantitative evaluation based on peptide counts revealed a small set of proteins enriched in patients. One of the most strongly accumulating proteins was clusterin, an extracellular chaperone with functional features similar to heat shock proteins (see below). Clusterin enrichment was confirmed by immunohistochemistry and shown to also occur in the cerebrovasculature of a CADASIL mouse model (Arboleda-Velasquez et al., 2011). Furthermore, immuno-electron microscopy analysis suggested its presence in human GOM deposits. An independent histological analysis of a larger number of post-mortem samples confirmed the clusterin enrichment in patient vessels, especially in white matter arteries, but a colocalization with GOM deposits could not be observed (Craggs et al., 2016). In the proteomic study by Nagatoshi et al. (2017) leptomeningeal arteries from two autopsied patients and the superficial temporal artery from one biopsied patient were used, but LC-MS/MS analysis yielded only a few proteins consistently detected in both the patient and control group. Label-free quantification revealed serum amyloid P component (SAP), annexin A2, and periostin as most strongly enriched proteins, but only SAP accumulation could be confirmed by immunohistochemistry. It was further shown to colocalize with Notch3^ECD^ aggregates in patient brain tissue sections and to interact with a recombinant Notch3^ECD^ fragment *in vitro* indicating involvement in GOM formation. The plasma protein SAP is a well-known universal constituent of human amyloid deposits and might be a general component of extracellular protein aggregates (see below) (Pepys, 2018).

**TABLE 1:** Summary of major proteins with increased abundance in proteomic SVD studies.

**Disease**	**Study**	**Tissue/vessel type**	**Proteins**					
			
			**SAP**	**TIMP3**	**VTN**	**APOE**	**CLU**	**HTRA1**
CADASIL	Arboleda-Velasquez et al., 2011	Autopsy, laser-microdissected subcortical arterioles	ND	ND	ND	ND	↑	ND
	Monet-Lepretre et al., 2013	Autopsy, enriched GOM from isolated brain vessels	↑	↑	↑	↑	↑	↑
	Nagatoshi et al., 2017	Autopsy, biopsy, laser-microdissected leptomeningeal	↑	=	↑	ND	=	=
		vessels						
	Zellner et al., 2018	Autopsy, isolated cortical and subcortical vessels	↑	↑	↑	↑	↑	↑
CAA	Manousopoulou et al., 2017	Autopsy, Isolated leptomeningeal vessels	↑	↑	↑	↑	↑	ND
	Inoue et al., 2017	Autopsy, laser-microdissected leptomeningeal vessels	=	=	↑	↑	↑	ND
	Hondius et al., 2018	Autopsy, laser-microdissected brain vessels	↑	ND	ND	↑	↑	↑
	Endo et al., 2019	Biopsy, laser-microdissected leptomeningeal and	ND	=	↑	↑	↑	ND
		cortical vessels						

*↑ Increased abundance = no significant abundance difference ND not detected or data not available. Proteins investigated in more detail in individual studies are highlighted. SAP, serum amyloid P component; TIMP3, tissue inhibitor of metalloproteinases 3; VTN, vitronectin; APOE, apolipoprotein E; CLU, clusterin; HTRA1, high temperature requirement A1 protease.*

In the study by Monet-Lepretre et al. (2013) the proteomics work on CADASIL was extended by establishing a method to isolate brain vessels from human autopsy material and by enriching GOM using a biochemical fractionation procedure. After nanoLC-MS/MS performed on material from one CADASIL patient and one control subject and subsequent quantification by peptide counting, a number of proteins with increased abundance were identified including clusterin and SAP. Enrichment was also observed for the two matrisomal proteins tissue inhibitor of metalloproteinases 3 (TIMP3) and vitronectin as well as for HTRA1, the serine protease genetically associated with CARASIL (see above). TIMP3 and vitronectin were shown to colocalize with GOM deposits, to bind to Notch3^ECD^ *in vitro* and to also accumulate in brain vessels of a CADASIL mouse model. From these and other studies (Kast et al., 2014; Lee et al., 2014; Zhang et al., 2015), it became increasingly clear, that the accumulation of ECM proteins represents a critical step in CADASIL pathogenesis likely causing a dysregulation of ECM homeostasis and multifactorial toxicity (Joutel et al., 2016). To provide further support for this hypothesis in a larger cohort and with increased proteome depth, Zellner et al. (2018) conducted label-free quantitative LC-MS/MS on brain vessels from six patients carrying five different Notch3 mutations and six age-matched healthy controls. For the first time, a truly quantitative comparison in combination with meaningful statistical evaluation of protein abundance changes in CADASIL was performed. An improved protocol of the brain vessel isolation technique published by Monet-Lepretre et al. (2013) was used, but no fractionation applied, resulting in the determination of a proteomic whole-vessel profile, in which matrisomal components were strongly overrepresented. The set of significantly enriched proteins included clusterin, TIMP3, vitronectin, SAP as well as HTRA1. Accumulation of HTRA1 was confirmed by quantitative immunoblotting, and its colocalization with Notch3^ECD^ deposits demonstrated by confocal immunofluorescence microscopy (Zellner et al., 2018). In addition, enrichment of several ECM proteins previously reported as HTRA1 substrates was noticed, suggesting reduced HTRA1 activity in the cerebrovasculature of CADASIL patients. To further substantiate this finding, the brain vessel proteome from HTRA1 knockout mice was determined to obtain a definite HTRA1 deficiency profile. Proteins accumulating in this profile were considered likely HTRA1 substrates and thus indicators for a loss of catalytic function. The comparison with the CADASIL profile indeed revealed a significant overlap which included clusterin, TIMP3, vitronectin and a number of other, mostly matrisomal proteins. Several previously uncharacterized, putative substrates were shown to be processed by HTRA1 in an *in vitro* assay (Zellner et al., 2018). These findings indicated the presence of a HTRA1 loss-of-function signature in the CADASIL vessel proteome and provided an unexpected mechanistical link between CADASIL and HTRA1-related SVDs. Proteomic analyses in CARASIL patients with genetically compromised HTRA1 activity have so far not been reported, likely due to the rareness of the disease. The discovery of affected heterozygous mutation carriers might facilitate the realization of such studies in the future (Verdura et al., 2015).

Proteomic approaches have also been applied in CAA to determine protein abundance changes associated with excessive vascular Aβ deposition (Table 1). Manousopoulou et al. (2017) performed quantitative LC-MS on leptomeningeal vessels isolated from autopsy tissue of four patients with severe arterial and capillary CAA. From the set of proteins with increased abundance, clusterin and TIMP3 were selected for immunohistochemistry analysis and shown to be enriched in leptomeningeal, but not cortical arteries and to largely colocalize with Aβ deposits. In the study by Inoue et al. (2017) laser capture microdissection was used to isolate vascular material from leptomeningeal arteries and neocortical arterioles from eight cases with severe CAA. Quantitative LC/MS-MS revealed enrichment of a variety of proteins including apolipoprotein E (APOE), vitronectin and clusterin. Sushi repeat-containing protein X-linked 1 (SRPX1), an apoptosis inducer in tumor cells previously described as SRPX, was investigated in more detail and shown in brain sections to co-accumulate with vascular Aβ deposits. SRPX mRNA levels were found to be increased in patient vessels and in primary cultures of cerebrovascular smooth muscle cells treated with Aβ peptides, indicating a transcriptional effect as cause of the observed enrichment (Inoue et al., 2017). SRPX was further shown to bind Aβ peptides *in vitro* and to enhance Aβ-induced caspase activity in cultured VSMCs. It was therefore proposed to promote Aβ-induced cerebrovascular degeneration in CAA. SRPX1 was not found to be enriched in most of the other proteomic studies and is therefore not further discussed.

The study by Hondius et al. (2018) investigated capillary (type I) CAA as a frequent comorbidity in AD and compared the proteomic profiles of microdissected gray matter material (including vessels) from AD cases with and without vascular amyloid pathology. After label-free quantification clusterin emerged as most significantly enriched protein in the CAA group extending the results of an earlier histological investigation (Verbeek et al., 1998). CAA-specific accumulation was also observed for norrin (NDP) and collagen α2(VI) (COL6A2) and colocalization with perivascular Aβ deposits demonstrated by immunohistochemistry. In contrast, APOE and SAP were found to be enriched in both the CAA and the AD group. HTRA1 was detected in none of the control samples and in only one AD sample, but in all CAA samples prompting the authors to claim its CAA-specific accumulation (Hondius et al., 2018). In partial agreement with this, immunohistochemistry staining associated with Aβ deposits was more intense in CAA than in AD tissue sections, a finding confirming the results of an earlier study (Grau et al., 2005). Vascular APOE and HTRA1 accumulation was also reported in Tg-SwDI mice, a CAA model carrying three human amyloid precursor protein (APP) mutations (Swedish, Dutch and Iowa) (Searcy et al., 2014). A quantitative comparison of the proteomic profiles of older mice exhibiting prominent vascular amyloid pathology with younger mice showing minimal Aβ deposition revealed APOE and HTRA1 as the proteins with the largest abundance increase. For both, the accumulation was confirmed by immunohistochemistry, but no clear colocalization with Aβ deposits could be observed. In the proteomic study by Endo et al. (2019) quantitative analysis was performed on biopsied, microdissected samples of leptomeningeal and cortical vessels obtained from six CAA patients who had underwent surgery for large lobar hemorrhages. Clusterin and APOE were most strongly enriched and their effects on Aβ aggregation investigated using an *in vitro* model of CAA recapitulating the intramural periarterial drainage process. Physiological concentrations of APOE and clusterin delayed the initiation time of Aβ aggregation kinetics in a concentration-dependent manner and thus, both were proposed to represent extracellular chaperones inhibiting vascular Aβ deposition.

## Extracellular Proteostasis in SVD

**FIGURE 1:**
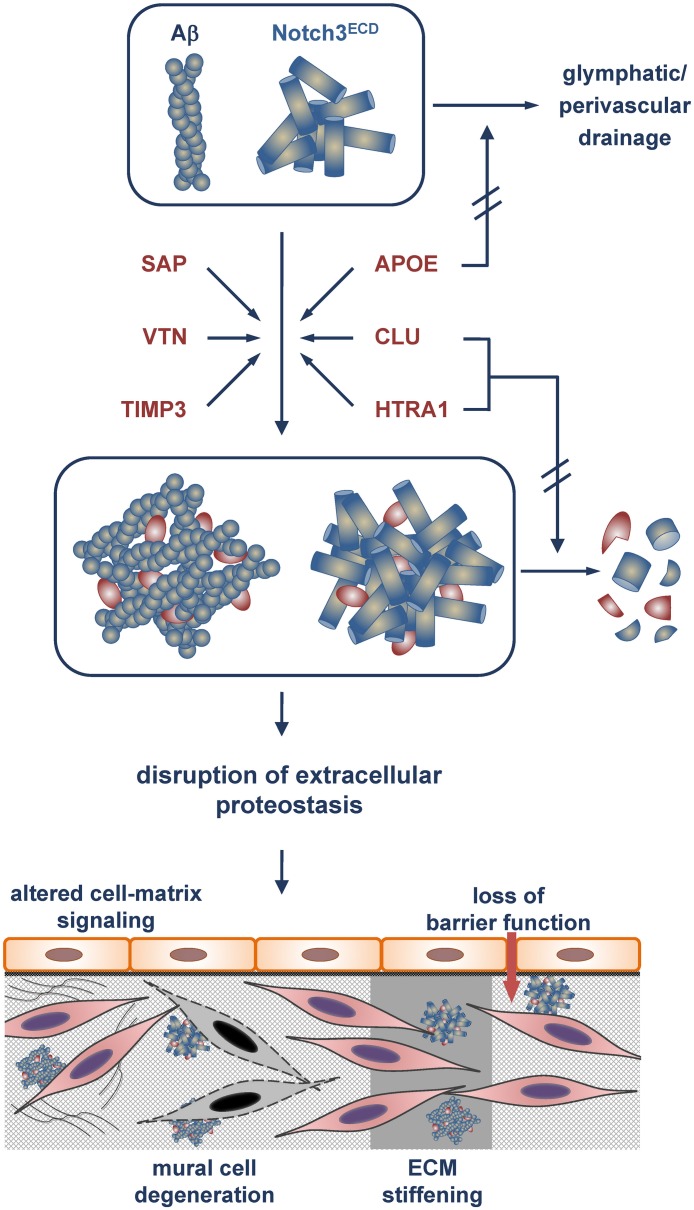
Model of Aβ and Notch3^ECD^ aggregate formation and proteostasis disruption in the brain vessel wall. Incorporation of the metastable proteins SAP, vitronectin (VTN) and TIMP3 with an increased tendency toward misfolding stabilizes Aβ and Notch3^ECD^ aggregates and/or accelerates aggregate growth. Excessive recruitment of the chaperones/clearance factors APOE, clusterin (CLU) and HTRA1 results in their depletion from the proteostasis network and a reduction or even loss of their activity. While APOE is impaired in its role in clearing of Aβ (and other misfolded proteins) from the brain (e.g., via glymphatic/perivascular pathways), clusterin and HTRA1 are compromised in their tasks to promote aggregate removal or degradation. The eventual disruption of extracellular proteostasis initiates a variety of pathophysiological processes including dysregulation of cell-matrix signaling, mural cell degeneration, alteration of physical ECM properties such as stiffness and loss of barrier function.

Despite the substantial differences in sensitivity, depth and quantification methodology of the described proteomic studies in CADASIL and CAA, they yielded a shared set of proteins showing enrichment with a relatively high consistency (Table 1). However, these proteins differ substantially in structure and function and are thus likely to contribute to disease pathogenesis in different ways (Figure 1). SAP, TIMP3 and vitronectin might associate with pathologic protein deposits primarily for structural reasons resulting in aggregate stabilization and/or growth. SAP is an abundant plasma protein of the pentraxin family and has gained a great deal of attention through its role as universal constituent of human amyloid deposits of all types including senile plaques in AD and vascular Aβ deposits in CAA (Pepys, 2018). It has been shown to avidly bind to and stabilize amyloid fibrils, possibly in an attempt to opsonize them for phagocytosis. Its depletion from the blood circulation was reported to result in the destabilization of amyloid deposits leading to the initiation of clinical trials in systemic amyloidosis and AD to investigate its therapeutic potential (Richards et al., 2015). SAP was also found to inhibit the heat-induced formation of amorphous aggregates, an activity believed to be relevant in protein misfolding during inflammatory processes (Ozawa et al., 2016). A role in non-amyloid protein aggregation is now further suggested by its accumulation observed in three of the four proteomic CADASIL studies as well as by its colocalization with Notch3^ECD^ deposits and its binding to Notch3^ECD^ *in vitro* (Nagatoshi et al., 2017).

Tissue inhibitor of metalloproteinases 3 (TIMP3) is a matrisomal protein well known for its role in ECM homeostasis. It controls the activity of proteases degrading matrix components and catalyzing the shedding of ectodomains from cell surface proteins (Arpino et al., 2015). Although TIMP3 enrichment was found in only one of the CAA studies, the presented immunohistochemistry results clearly support the conclusion of Aβ-associated accumulation (Manousopoulou et al., 2017). Similarly, TIMP3 was shown to colocalize with Notch3^ECD^ deposits in CADASIL-affected vessels (Monet-Lepretre et al., 2013). Moreover, increased TIMP3 levels were also observed in a CADASIL mouse model and follow-up experiments suggested an impairment of a signaling cascade regulating arterial smooth muscle cell activity leading to cerebral blood flow deficits (Capone et al., 2016b), a symptom known to occur early in CADASIL pathogenesis. Genetic mutations in the *TIMP3* gene result in Sorsby fundus dystrophy (SFD) (Anand-Apte et al., 2019), a rare, dominantly inherited, adult-onset retinal dystrophy clinically and histopathologically related to age-related macular degeneration, the most common cause of blindness in adults. SFD is characterized by the presence of drusen, extracellular deposits located below the basement membrane of the retinal pigment epithelium. TIMP3 shows a striking structural similarity to Notch3 in that its primary sequence contains an even number of cysteines all forming disulfide bonds (Anand-Apte et al., 2019). Very similar to CADASIL mutations SFD-causing variants are typically missense mutations altering the number of cysteine residues and enhancing TIMP3 multimerization/aggregation. It is thus conceivable that the recruitment of TIMP3 to Aβ and Notch3^ECD^ aggregates promotes TIMP3 misfolding resulting in a further enhancement of the aggregation process. Also vitronectin is a well-known ECM component and a major adhesive protein of the extracellular environment (Leavesley et al., 2013). It interacts with a multitude of proteins and promotes cell adhesion, spreading, and migration. Its accumulation in three of the four proteomic CAA studies extends previous histological studies which showed enrichment in the senile plaques of AD patients (Shin et al., 2008; Winter et al., 2015). Vitronectin was furthermore reported to be able to form spherical oligomers as well as typical amyloid fibrils *in vitro* which are toxic for cultured cells (Shin et al., 2008). Its role in Notch3^ECD^ aggregation has not been investigated yet, but its genetic reduction in a CADASIL mouse model led to a reversal of white matter pathology, although the underlying molecular mechanisms remained unclear (Capone et al., 2016a). Thus, SAP, TIMP3 and vitronectin might represent metastable proteins, whose tendency toward misfolding and multimerization is enhanced upon recruitment to Notch3^ECD^ or Aβ deposits.

In contrast, APOE, clusterin and HTRA1 accumulation in CADASIL and CAA is likely to follow a different mechanism due to their established or presumed ability to act as molecular chaperones. APOE has long been known for its role in modulating AD and CAA risk (depending on the combination of the three human isoforms APOE2, APOE3 and APOE4) and for its ability to bind Aβ peptides and to be recruited to amyloid deposits (Yu et al., 2014). It has been suggested to modulate Aβ deposition in a variety of ways: (1) acceleration of Aβ aggregation and deposition, (2) facilitation of Aβ uptake by microglia, astrocytes and neurons, (3) enhancement of enzymatic Aβ degradation, and (4) facilitation of Aβ clearance via transport across the blood-brain barrier or by drainage via the interstitial fluid or the perivasculature. The identification of APOE as major accumulating factor in all the proteomic CAA studies as well as in the Tg-SwDI mouse model extends previous histological analyses (Verbeek et al., 1998) and provides further support for an important role of APOE in CAA pathogenesis. Some insight into a potential mechanism was gained in the study by Endo et al. (2019) in which an *in vitro* model of CAA recapitulating the intramural periarterial drainage pathway was used and an inhibitory role of APOE on the early phase of vascular Aβ deposition reported. The impact of APOE on Notch3^ECD^ in CADASIL is more difficult to assess, since no follow-up studies have been published to date. The influence of the APOE genotype on clinical CADASIL features was investigated in two studies, but while in one increased white matter hyperintensity volumes in *APOE2*, but not *APOE4* carriers were reported (Gesierich et al., 2015), in the other no association with MRI lesion volumes or disease phenotype (age of onset, presence of stroke) could be detected (Singhal et al., 2004). Interestingly, two studies demonstrated proteolytic APOE processing by HTRA1 (Chu et al., 2016; Munoz et al., 2018) providing a potential link to HTRA1-related SVDs.

Clusterin is a ubiquitously and constitutively expressed protein present in a wide range of tissues and body fluids (Wilson and Zoubeidi, 2017). It is involved in a number of cellular processes including inhibition of the complement system, lipid transport, regulation of cell survival and cell death pathways and has often been referred to as an extracellular chaperone. Its accumulation reported in all four CAA proteome studies is in agreement with earlier histological findings (Verbeek et al., 1998; Craggs et al., 2016) and its well-established link to AD (Foster et al., 2019). Clusterin is able to bind a wide range of Aβ oligomers (from dimers to 50-mers) and to interfere with Aβ peptide aggregation and amyloid fibril formation *in vitro* (Narayan et al., 2012). It further promotes Aβ clearance in a variety of experimental settings (Wojtas et al., 2017; Endo et al., 2019). From these observations, an anti-amyloidogenic role of clusterin due to its chaperone function was deduced, but studies on clusterin knockout mice have not provided clear answers on its neuroprotective function and fueled the ongoing controversy about its beneficial role in amyloid diseases (Foster et al., 2019). The detection of clusterin enrichment in three of the four proteomic CADASIL studies suggests a role also in non-amyloid protein aggregation. However, conflicting results have been reported about its precise colocalization with Notch3^ECD^ aggregates. Whereas Arboleda-Velasquez et al. (2011) reported the presence of clusterin in GOM deposits, Craggs et al. (2016) were unable to confirm this finding and even showed a clear separation of clusterin and Notch3 immunofluorescence staining patterns in patient vessels. They nevertheless observed a correlation between clusterin levels and white matter pathology scores, not only in CADASIL, but also in other leukoencephalopathies, suggesting a more general role of clusterin in ischemic SVD.

The proteomic results on the serine protease HTRA1 might have the most far-reaching impact on our understanding of SVD pathomechanisms. Its involvement in cerebrovascular disease had first become clear with the discovery of its genetic association with monogenic recessive and dominant hereditary SVD forms (Hara et al., 2009; Verdura et al., 2015). The realization of the loss-of-function nature of pathogenic HTRA1 mutations indicated a critical role of its catalytic activity for normal cerebrovascular function. However, the substrates involved in SVD pathogenesis as well as the molecular processes affected by HTRA1 deficiency including a possible dysregulation of TGFβ signaling have not yet been clearly determined (Hara et al., 2009; Shiga et al., 2011; Beaufort et al., 2014). The accumulation of HTRA1 in CADASIL and the high degree of colocalization with Notch3^ECD^ deposits *in vivo* provided further support of its key role in the brain microvasculature (Monet-Lepretre et al., 2013; Zellner et al., 2018). Moreover, the phenotypic similarities between CADASIL and the HTRA1-related SVDs as well as the extensive overlap between the proteomic CADASIL signature and the murine HTRA1 loss-of-function profile strongly suggest a role of HTRA1-mediated substrate processing also in CADASIL (Monet-Lepretre et al., 2013; Zellner et al., 2018). APOE, vitronectin and TIMP3 have all been shown in earlier studies to be processed by HTRA *in vitro*, and for APOE even isoform-specific differences were reported, with APOE4 more efficiently cleaved compared to APOE3 (An et al., 2010; Chu et al., 2016; Munoz et al., 2018). Thus, a loss in HTRA1 catalytic activity might contribute to the accumulation of these proteins under pathophysiological conditions. A link between HTRA1 and Aβ metabolism was first indicated by the demonstration of HTRA1-mediated degradation of Aβ peptides *in vitro* and the colocalization of HTRA1 with parenchymal and perivascular Aβ deposits (Grau et al., 2005). In subsequent studies, HTRA1 was shown to also degrade tau protein in monomeric and fibrillar form (Tennstaedt et al., 2012). The degradation of tau fibrils was furthermore reported to involve the disintegration of the fibrillar core structure and the subsequent solubilization of fibrils allowing productive interaction of aggregated polypeptides with the active site of HTRA1 for rapid degradation (Poepsel et al., 2015). Thus, HTRA1 apparently combines chaperone and protease activity in a single polypeptide and might be able to efficiently dissolve and degrade pathological protein aggregates thereby antagonizing neurodegeneration caused by excessive protein aggregation (Shorter, 2017). The proteomic findings in CAA patients and the Tg-SwDI mouse model, most importantly those of the Hondius et al. (2018) study, showing preferential HTRA1 accumulation in vascular Aβ deposits, suggest that its function might especially be important in cerebrovascular Aβ biology.

## Conclusion

Proteomic studies on human cerebrovascular tissue have identified several extracellular proteins accumulating with unexpected consistency in CADASIL and CAA, two neurovascular conditions caused by protein misfolding and aggregation. The enrichment of the same set of proteins in two different types of protein deposits suggests the contribution of shared pathways to pathogenesis. A search for these proteins within the mouse brain vasculome, a previously determined transcriptome of cerebral endothelial cells (Guo et al., 2012), revealed only a marginal overlap, arguing against this cell type as a major source of the accumulating proteins. Although the molecular details of cerebrovascular protein aggregation are still largely unknown, the identified proteins might facilitate disease progression by two different mechanisms (Figure 1). SAP, TIMP3 and vitronectin appear to be incorporated into preexisting protein deposits mainly due to their metastability, thus contributing directly to aggregate stabilization or growth and exerting a gain-of-toxic-function effect. In contrast, APOE, clusterin and HTRA1 are possibly actively recruited to pathological protein aggregates due to their chaperone function within the extracellular proteostasis network. Under physiological conditions they facilitate the refolding of misfolded proteins or promote their removal either by degradation or by clearance via vascular routes. The permanent encroachment of chaperone capacity under pathophysiological conditions, e.g., during aging or due to genetic mutations, will inevitably result in a network overload. This process is aggravated by the excessive recruitment of chaperones to protein deposits resulting in their sequestration and depletion, tantamount to a loss-of-function mechanism (Balchin et al., 2016). Thus, cerebrovascular disease might be the result of a combination of a gain-of-toxic-function and loss-of-function effect, similar to scenarios discussed in neurodegenerative disorders such as Parkinson’s disease (Winklhofer et al., 2008; Benskey et al., 2016). The consequences of a disruption of extracellular proteostasis on the functionality of cerebral vessels are largely unknown, but they are likely to include the dysregulation of cell-matrix signaling, the degeneration of mural cells, alterations of physical ECM properties such as stiffness, and loss of barrier function (Figure 1). Whether impaired extracellular proteostasis might also contribute to pathologies of the brain microvasculature not associated with excessive protein aggregation such as arteriolosclerosis is currently difficult to assess, largely due to the lack of appropriate proteomic studies. While substantial alterations in the vessel architecture are a key pathological finding in sporadic SVD, a causal link to disrupted proteostasis has so far not been established. The further elucidation of proteostasis mechanisms will undoubtedly advance our understanding of the physiological and pathophysiological processes underlying brain health and disease.

## Author Contributions

CH wrote the manuscript and prepared the figure.

## Conflict of Interest

The author declares that the research was conducted in the absence of any commercial or financial relationships that could be construed as a potential conflict of interest.
